# Supplementary material for: Plasmodium falciparum var genes expressed in children with severe malaria encode CIDRα1 domains
Source: EMBO Mol Med. 2016 Jun 27;8(8):839–50. doi: 10.15252/emmm.201606188 (PMC4967939; doi:10.15252/emmm.201606188)
Supplement: Supplementary file 1 — Appendix [file EMMM-8-839-s001.pdf]

Appendix

Contents:

Table S1
Table S2
Figure S1
Figure S2
Figure S3

Table S1: Distribution (%) of *var* transcripts by DBLα domain type

	**Uncomplicated (*n*=8)**	**Severe malaria (*n*=36)**	**P§**	**Severe anaemia (*n*=21)**	**Cerebral malaria (*n*=9)**	**P§**
Domain subtypes	Median [IQR]	Median [IQR]		Median [IQR]	Median [IQR]	
DBLα1	9 [0 - 49]	23 [11- 57]	0.32	20 [7 - 57]	57 [21- 77]	0.10
DBLα1.[1/2/4/7]	4 [0 - 9]	19 [9 - 57]	0.01	18 [4- 44]	57 [14- 8]	0.12
DBLα1.5	0 [0 - 0]	0 [0 - 0]	0.22	0 [0 - 0]	0 [0 - 0]	0.51
DBLα1.6	0 [0 - 0]	0 [0 - 0]	0.22	0 [0 - 0]	0 [0 - 0]	0.13
DBLα1.8	0 [0 - 0]	0 [0 - 0]	1.00	0 [0 - 0]	0 [0 - 0]	0.44
DBLα2	0 [0 - 24]	13 [0 - 39]	0.24	16 [0 - 42]	0 [0 - 19]	0.21
DBLα0	43.7 [21.9 - 84.7]	20.8 [0 - 35.1]	0.05	14.1 [0 - 32.1]	23.0 [5.4 - 25.8]	0.80
VAR1	0 [0 - 2]	0 [0 - 0]	1.00	0 [0 - 0]	0 [0 - 0]	0.83
DBLα1.[1/2/4/7] +DBLα2 + DBLα2 prediction*	9 [4 - 42]	65 [28- 83]	0.005	68 [28- 84]	63 [38- 68]	0.74
DBLα1.[1/2/4/7] : (DBLα2+DBLα2–prediction*) ratio$	1.0 [0.2–8.9]	0.9 [0.3 - 13.6]	0.87	0.6 [0.2 - 3.2]	7.0 [0.7 - 66.6]	0.09

§ P values were calculated using Wilcoxon rank sum test

* Transcripts annotated with a DBLα1 or DBLα2 domain or predicted by the DBLα tag sequence to encode a DBLα2 domain.

$ The patient-wise ratio between the level of transcripts encoding group A type DBLα domains (DBLα1.[1/2/4/7]) and group B type DBLα domains (DBLα2) coupled to CIDRα1.[4-7] and CIDRα1.[1/8] types, respectively, was calculated as follows: (∑DBLα1.[1/2/4/7] + 0.01) : (∑DBLα2/α2-prediction+0.01).

Table S2: Proportions (%) of *var* transcripts encoding the DBL domains listed

	**Uncomplicated (*n*=8)**	**Severe malaria (*n*=36)**	P§
Domain subtypes#	Median [IQR]	Median [IQR]	
DBL2β	49 [20 - 65]	66 [40 - 79]	0.17
DBL2β1	0 [0 - 0]	0 [0 - 14]	0.04
DBL2β3	0 [0 - 34]	12 [0 - 26]	0.74
DBL2β5	0 [0 - 26]	0 [0 - 11]	0.57
DBL2β12	0 [0 - 4]	4 [0 - 30]	0.13
DBL2β A type*	19 [0 - 47]	48 [23 - 75]	0.05
DBL2β BC type$	5 [0 - 41]	0 [0 - 16]	0.31
DBL2γ	0 [0 - 3]	0 [0 - 4]	1
DBL2δ	34 [6 - 41]	7 [0 - 22]	0.05
DBLε/ζ	11 [6 - 62]	4 [0 - 20]	0.13

# The number before the Greek letter reflects that the domain is the second DBL domain encoded by the gene.
§ P values were calculated using Wilcoxon rank sum test.
* DBL2β A type: DBLβ [1/3/6/7/9/11/12]. DBLβ12 is part of the group B/A chimeric DC8 PfEMP1; DBLβ12 was here considered a group A domain.
$DBLβBC types: DBLβ[2/4/5/8/10/13]

Figure S1. Annotation of *var* genes expressed in patients suffering from malaria

Patient ID and clinical symptoms/grouping cerebral malaria (CM), severe anaemia (SA), hyper-parasitaemia (hyper), respiratory distress (RD) or uncomplicated malaria (UM) is shown. Patients who died are also indicated (┼). Domain composition of the six most abundant *var* transcripts in each patient is shown. For some genes the long range PCR amplification was not successful and these genes are annotated based on the DBLα-tag sequence (*Group A, Group B/C, DBLα2 prediction* or *VAR1*). Transcript proportions are here given as the un-normalized proportion of the total DBLα-tag sequence read count within the patient.

Patient	Syndrome(s)	Haemoglobin (g/dL)	Blantyre score	Gene	Proportion	D1	D2	D3	D4	D5	D6	D7	D8	D9	
1734	SA RD	3.7	5	1734-1	11%	DBLα0.11	CIDRα2.1	DBLβ5	DBLδ1	CIDRγ11	Intron				
				1734-2	9%	DBLα0.13	CIDRα2.8	...							
				1734-3	7%	Group B/C									
				1734-4	6%	DBLα0.15	CIDRα2.10	DBLδ1	CIDRγ7	...					
				1734-5	5%	DBLα0.4	CIDRα6	DBLβ5	DBLγ12	DBLδ7	CIDRγ6	DBLζ1	DBLε14	Intron	
				1734-6	4%	Group B/C									
1841	SA	4.9	5	1841-1	26%	DBLα1.2	CIDRα1.5a	DBLβ6	DBLγ2	DBLγ4	DBLδ1	CIDRβ1	Intron		
				1841-2	15%	DBLα2	CIDRα1.8a	DBLβ12	DBLζ3	DBLε6	DBLδ1	CIDRβ1	Intron		
				1841-3	14%	DBLα2	CIDRα1.1	DBLβ12	DBLγ4	DBLδ1	CIDRγ11	Intron			
				1841-4	11%	DBLα2 prediction									
				1841-5	9%	Group A									
				1841-6	6%	Group A									
1861	SA	4.7	5	1861-1	36%	Group B/C									
				1861-2	31%	DBLα2 prediction									
				1861-3	8%	DBLα0.21	CIDRα2.1	DBLδ1	CIDRγ12	Intron					
				1861-4	6%	DBLα0.8	CIDRα3.1	DBLβ5	DBLγ10	DBLδ1	CIDRβ6	Intron			
				1861-5	6%	Group B/C									
				1861-6	4%	Group A									
1869	SA	2.2	5	1869-1	10%	DBLα0.1	CIDRα3.2	DBLδ1	CIDRβ1	Intron					
				1869-2	8%	DBLα0.21	CIDRα2.1	DBLβ2	DBLδ1	CIDRβ6	DBLγ3	DBLζ4	Intron		
				1869-3	8%	DBLα2	CIDRα1.8a	DBLβ3	DBLγ11	DBLζ4	Intron				
				1869-4	8%	DBLα1.1	CIDRα1.7	DBLβ3	DBLγ2	DBLγ4	DBLγ2	DBLδ1	CIDRβ6	Intron	
				1869-5	8%	DBLα1.4	CIDRα1.3	DBLβ1	DBLγ15	DBLε1	DBLγ8	DBLζ1	DBLε5	Intron	VAR1
				1869-6	6%	Group B/C									
1920	SA †	4.8	4	1920-1	59%	DBLα2	CIDRα1.1	DBLβ12	DBLγ6	DBLδ4	CIDRδ1	Intron			
				1920-2	10%	DBLα1.7	CIDRα1.4	DBLβ3	DBLγ2	DBLγ4	Intron				
				1920-3	9%	DBLα2 prediction									
				1920-4	5%	Group A									
				1920-5	3%	Group A									
				1920-6	3%	Group A									
1922	SA	3.7	5	1922-1	31%	Group B/C									
				1922-2	21%	DBLα0.19	CIDRα6	DBLδ1	CIDRβ1	Intron					
				1922-4	16%	DBLα0.19	...								
				1922-3	15%	DBLα0.6	CIDRα3.1	DBLβ5	DBLγ13	DBLδ1	CIDRβ1	Intron			
				1922-5	12%	DBLα0.5	CIDRα2.6	DBLγ2	DBLδ1	CIDRβ1	...				
				1922-6	3%	Group B/C									
1931	SA	3.8	5	1931-1	24%	DBLα0.15	CIDRα3.1	DBLβ5	DBLγ9	DBLδ1	CIDRγ9	DBLε2	DBLζ3	DBLε6	Intron
				1931-2	21%	DBLα2 prediction									
				1931-3	10%	DBLα0.6	CIDRα3.1	DBLγ5	DBLδ1	CIDRβ1	Intron				
				1931-4	8%	DBLα0.13	CIDRα2.3	DBLβ3	DBLγ5	DBLδ1	CIDRβ5	Intron			
				1931-5	7%	DBLα2	CIDRα1.1	DBLβ3	DBLγ13	DBLζ2	DBLε4	Intron			
				1931-6	6%	Group A									
1939	SA	4.1	5	1939-1	24%	DBLα1.7	CIDRα1.4	DBLβ1	DBLγ10	DBLδ1	CIDRβ5	Intron			
				1939-2	22%	DBLα2	CIDRα1.1	DBLβ12	DBLγ6	DBLγ4	DBLζ5	...			
				1939-3	12%	DBLα1.1	CIDRα1.4	DBLβ12	DBLγ2	DBLγ4	DBLδ1	CIDRβ5	Intron		
				1939-4	7%	DBLα1.7	CIDRα1.4	DBLβ1	DBLβ6	DBLγ2	DBLγ11	DBLδ1	CIDRβ1	Intron	
				1939-5	6%	DBLα2	CIDRα1.8b	DBLβ12	DBLγ4	DBLδ1	CIDRβ6	...			
				1939-6	5%	Group A									
1965	SA	4.4	5	1965-1	30%	DBLα2	CIDRα1.8a	DBLβ12	DBLγ4	DBLδ4	CIDRδ1	...			
				1965-3	27%	DBLα2	CIDRα1.8a	DBLβ12	DBLγ4	...					
				1965-5	11%	DBLα1.2	CIDRα1.5b	DBLβ3	DBLγ17	DBLγ14	DBLζ5	DBLε4	Intron		
				1965-2	9%	DBLα1.2	CIDRα1.5a	DBLγ17	DBLδ5	CIDRβ4	DBLβ6	DBLγ13	Intron		
				1965-7	6%	DBLα1.2	CIDRα1.6b	DBLβ3	DBLγ12	DBLδ5	CIDRβ4	DBLε2	...		
				1965-11	5%	Group A									
1971	SA	3.3	5	1971-1	21%	DBLα0.5	CIDRα2.5	DBLδ1	CIDRβ1	DBLζ6	DBLε6	Intron			
				1971-2	14%	Group A									
				1971-3	12%	DBLα2	CIDRα1.8a	DBLβ12	DBLζ3	DBLε6	DBLδ1	CIDRβ1	Intron		
				1971-4	12%	DBLα1.2	CIDRα1.4	DBLβ7	DBLγ13	DBLδ7	CIDRβ4	DBLβ7	DBLγ2	...	
				1971-5	11%	DBLα2	CIDRα1.1	DBLβ12	DBLγ4	DBLδ1	CIDRγ11	Intron			
				1971-6	6%	Group A									
2021	SA	4.4	5	2021-1	21%	DBLα0.13	CIDRα2.3	DBLδ1	CIDRγ5	Intron					
				2021-2	9%	DBLα1.1	CIDRα1.7	DBLβ1	DBLγ16	DBLγ2	DBLδ1	CIDRβ1	Intron		
				2021-3	8%	DBLα0.9	CIDRα2.7	DBLδ1	CIDRβ1	Intron					
				2021-5	7%	DBLα0.19	CIDRα3.4	...							
				2021-4	6%	DBLα0.18	CIDRα4	DBLβ5	DBLδ1	...					
				2021-6	5%	Group B/C									
2052	SA	3.4	5	2052-1	21%	DBLα1.8	CIDRβ4	DBLγ2	DBLγ11	Intron					
				2052-2	17%	DBLα2	CIDRα1.1	DBLβ12	DBLγ6	DBLδ1	CIDRβ1	Intron			
				2052-3	13%	DBLα1.8	CIDRβ2	DBLγ7	DBLε11	DBLζ3	DBLε6	Intron			
				2052-4	7%	DBLα0.9	CIDRα2.2	DBLδ1	CIDRβ1	Intron					
				2052-5	6%	DBLα1.1	CIDRα1.2	DBLβ11	DBLγ1	DBLε1	DBLγ8	DBLζ1	DBLε5	Intron	VAR1
				2052-6	6%	Group B/C									
2083	SA	4.1	5	2083-1	21%	DBLα1.1	CIDRα1.7	DBLβ1	DBLγ2	DBLγ4	DBLγ2	DBLζ3	DBLε6	Intron	
				2083-2	13%	DBLα1.2	CIDRα1.7	DBLβ3	DBLβ6	DBLγ13	DBLδ1	CIDRβ6	Intron		
				2083-3	13%	DBLα2	CIDRα1.8a	DBLβ12	DBLγ17	DBLδ1	CIDRβ5	Intron			
				2083-4	13%	DBLα2	CIDRα1.1	DBLβ1	DBLγ16	DBLγ14	...				
				2083-5	10%	DBLα1.2	CIDRα1.6a	DBLβ12	DBLγ6	DBLγ2	DBLδ1	CIDRβ6	Intron		
				2083-6	7%	Group A									
2110	SA RD	1.7	5	2110-1	39%	DBLα1.2	CIDRα1.5a	DBLβ9	DBLε13	DBLγ11	DBLδ7	CIDRβ4	Intron		
				2110-2	15%	DBLα0.18	CIDRα6	DBLβ5	DBLδ1						
				2110-3	15%	DBLα2	CIDRα1.1	DBLβ12	DBLγ6	DBLδ1	CIDRβ5	Intron			
				2110-4	12%	VAR1									
				2110-5	7%	Group A									
				2110-6	6%	Group A									

Patient	Syndrome(s)	Haemoglobin (g/dL)	Blantyre score	Gene	Proportion	D1	D2	D3	D4	D5	D6	D7	D8	D9	
2114	SA	3.8	5	2114-1	25%	DBLα0.11	CIDRα2.4	DBLδ1	CIDRβ1	Intron					
				2114-2	19%	DBLα1.1	CIDRα1.7	DBLβ11	DBLγ2	DBLγ9	DBLδ1	CIDRβ3	Intron		
				2114-3	16%	DBLα0.9	...								
				2114-4	16%	Group A									
				2114-5	8%	DBLα1.5	CIDRδ1	DBLβ3	DBLγ12	DBLγ2	Intron				
				2114-6	3%	Group A									
2120	SA	3.9	5	2120-1	89%	DBLα2 prediction									
				2120-2	9%	DBLα1.7	CIDRγ3	DBLβ6	DBLγ2	DBLγ11	...				
				2120-3	2%	DBLα1.4	CIDRα1.3	DBLβ1	DBLγ15	DBLε1	DBLγ8	DBLζ2	DBLε5	Intron	VAR1
2121	SA	4.5	5	2121-1	19%	Group A									
				2121-2	14%	DBLα2	CIDRα1.1	DBLβ1	DBLγ4	DBLδ1	CIDRβ6	Intron			
				2121-3	9%	Group A									
				2121-4	6%	DBLα0.11	CIDRα2.4	DBLδ1	CIDRβ1	Intron					
				2121-5	5%	Group A									
				2121-6	5%	Group A									
2132	SA RD	4.4	3	2132-1	23%	DBLα1.7	CIDRα1.4	DBLβ3	DBLβ7	DBLδ1	CIDRγ9	Intron			
				2132-2	17%	DBLα1.7	CIDRα1.4	DBLβ1	DBLγ13	DBLγ12	DBLδ5	CIDRβ4	DBLβ9	DBLγ9	Intron
				2132-3	10%	DBLα1.1	CIDRα1.7	DBLβ1	DBLγ2	DBLγ4	DBLγ2	DBLζ3	DBLε6	Intron	
				2132-4	7%	DBLα0.1	CIDRα3.4	...							
				2132-5	5%	DBLα1.2	CIDRα1.1	DBLβ12	DBLγ11	DBLγ6	Intron				
				2132-6	4%	Group B/C									
2142	SA	3.4	5	2142-1	42%	DBLα2	CIDRα1.1	DBLβ12	DBLγ4	DBLδ1	CIDRβ1	Intron			
				2142-2	12%	DBLα0.6	CIDRα3.4	DBLβ5	DBLγ16	DBLδ1	CIDRβ1	Intron			
				2142-3	11%	DBLα2	CIDRα1.1	DBLβ12	DBLγ4	DBLδ1	CIDRβ1	Intron			
				2142-4	7%	DBLα2	CIDRα1.8a	DBLβ12	DBLγ4	DBLζ3	DBLε12	Intron			
				2142-5	5%	DBLα1.4	CIDRγ3	DBLβ6	DBLγ2	DBLγ12	DBLδ1	CIDRβ1	Intron		
				2142-6	5%	Group A									
2157	SA RD	3.8	3	2157-1	17%	DBLα1.8	CIDRβ2	DBLγ7	DBLε11	DBLζ2	DBLε6	Intron			
				2157-2	17%	DBLα1.7	CIDRα1.4	DBLβ3	DBLγ2	DBLγ12	DBLδ5	CIDRβ5	Intron		
				2157-3	15%	DBLα2	CIDRα1.8b	DBLβ12	DBLβ5	DBLδ1	CIDRβ1	Intron			
				2157-4	7%	Group A									
				2157-5	6%	DBLα2 prediction									
				2157-6	5%	Group B/C									
2268	SA	4.5	5	2268-1	59%	DBLα2	CIDRα1.1	DBLβ3	DBLγ14	DBLζ5	DBLε4	Intron			
				2268-2	21%	DBLα1.4	CIDRα1.3	DBLβ1	DBLγ15	DBLε1	DBLγ8	DBLζ2	DBLε5	Intron	VAR1
				2268-3	4%	Group B/C									
				2268-4	3%	Group B/C									
				2268-5	3%	Group B/C									
				2268-6	3%	Group B/C									
1873	CM RD	N/A	0	1873-1	42%	Group B/C									
				1873-4	23%	DBLα2	CIDRα1.1	DBLβ12	DBLγ13	DBLδ1	CIDRβ1	Intron			
				1873-2	19%	DBLα2	CIDRα1.1	DBLβ3	DBLγ16	DBLδ5	CIDRβ4	DBLβ7	DBLγ12	Intron	
				1873-3	9%	DBLα0.13	CIDRα2.3	DBLδ1	CIDRβ7	Intron					
				1873-5	4%	VAR1									
				1873-6	2%	Group B/C									
1890	Hyper	6.8	5	1890-1	24%	DBLα0.11	CIDRα2.4	DBLδ1	CIDRβ1	Intron					
				1890-2	18%	DBLα1.2	CIDRα1.5a	DBLβ3	DBLγ12	DBLδ5	CIDRβ4	DBLβ9	DBLγ6	Intron	
				1890-3	15%	DBLα2	CIDRα1.1	DBLβ5	DBLδ1	CIDRβ1	Intron				
				1890-4	10%	DBLα2 prediction									
				1890-5	5%	DBLα1.7	CIDRα1.4	DBLβ3	DBLβ3	DBLγ11	...				
				1890-6	3%	Group A									
1914	CM Hyper	6.9	2	1914-21	37%	DBLα1.7	CIDRα1.4	DBLβ1	DBLβ6	DBLγ11	DBLδ1	CIDRγ9	Intron		
				1914-14	26%	DBLα1.7	CIDRα1.7	DBLβ6	DBLγ2	DBLγ6	DBLδ4	CIDRδ1	Intron		
				1914-12	22%	DBLα1.8	CIDRγ3	DBLγ7	DBLδ5	CIDRβ3	DBLβ9	DBLγ11	...		
				1914-20	4%	DBLα0.10	CIDRα2.2	DBLδ1	CIDRγ11	Intron					
				1914-16	2%	Group B/C									
				1914-27	2%	Group B/C									
1918	CM	N/A	0	1918-1	26%	DBLα1.2	CIDRα1.5a	DBLβ7	DBLγ10	DBLγ11	DBLγ4	DBLδ1	CIDRβ4	...	
				1918-2	17%	DBLα1.7	CIDRα1.4	DBLβ3	DBLγ11	DBLγ2	DBLδ1	CIDRβ1	Intron		
				1918-5	9%	DBLα1.2	CIDRα1.5b	DBLγ17	DBLδ5	CIDRβ3	DBLβ7	DBLγ2	Intron		
				1918-3	9%	DBLα1.2	CIDRα1.4	DBLβ1	DBLβ7	DBLγ13	DBLδ6	CIDRβ2	Intron		
				1918-4	8%	DBLα2	CIDRα1.1	DBLβ12	DBLγ11	DBLδ1	CIDRβ1	Intron			
				1918-6	4%	Group A									
1919	CM SA RD Hyper	3.4	2	1919-1	59%	DBLα2	CIDRα1.1	DBLβ12	DBLγ6	DBLδ4	...				
				1919-2	21%	DBLα1.7	CIDRα1.4	DBLβ3	DBLγ2	DBLγ4	Intron				
				1919-3	10%	DBLα1.7	CIDRα1.7	DBLβ1	DBLγ11	DBLδ1	CIDRγ9	Intron			
				1919-4	7%	DBLα2	CIDRα1.1	DBLβ12	DBLγ4	DBLγ14	DBLζ5	DBLε4	Intron		
				1919-5	3%	Group A									
1945	CM	7.5	0	1945-2	14%	Group A									
				1945-1	12%	DBLα1.2	CIDRα1.7	DBLβ3	DBLγ2	DBLγ11	DBLγ2	DBLδ1	CIDRβ1	Intron	
				1945-4	8%	DBLα0.1	...								
				1945-3	8%	DBLα1.8	CIDRγ3	DBLγ7	DBLζ5	DBLε11	DBLε12	Intron			
				1945-5	8%	DBLα2	CIDRα1.8b	DBLβ12	DBLγ4	DBLδ1	CIDRβ5	Intron			
				1945-6	7%	Group A									
1950	CM †	6.1	2	1950-1	30%	DBLα1.2	CIDRα1.5a	DBLβ6	DBLγ11	...					
				1950-2	9%	DBLα1.2	CIDRα1.6b	DBLβ3	DBLγ14	DBLδ1	CIDRβ1	Intron			
				1950-3	6%	DBLα0.24	CIDRα6	DBLδ1	CIDRβ1	Intron					
				1950-4	5%	Group B/C									
				1950-5	5%	Group B/C									
				1950-6	4%	Group B/C									
1974	CM	8.8	2	1974-1	14%	DBLα1.6	CIDRγ3	DBLγ11	DBLδ5	CIDRβ3	DBLβ6	Intron			
				1974-2	13%	DBLα0.11	CIDRα2.4	DBLδ1	CIDRβ1	DBLγ10	Intron				
				1974-3	11%	DBLα1.7	CIDRα1.4	DBLβ3	DBLβ7	DBLγ2	DBLγ17	DBLδ1	CIDRβ1	Intron	
				1974-4	9%	DBLα1.1	CIDRα1.7	DBLβ1	DBLγ6	DBLγ11	DBLδ1	CIDRβ1	Intron		
				1974-5	8%	DBLα1.7	CIDRα1.4	DBLβ3	DBLγ11	DBLγ11	DBLγ6	DBLδ1	CIDRβ1	Intron	
				1974-6	6%	Group B/C									

Patient	Syndrome(s)	Haemoglobin (g/dL)	Blantyre score	Gene	Proportion	D1	D2	D3	D4	D5	D6	D7	D8	D9	
1983	CM SA RD	5	1	1983-4	10%	DBLα1.1	CIDRα1.5a	DBLβ3	DBLγ12	DBLδ5	CIDRβ3	DBLβ7	DBLγ2	Intron	
				1983-3	10%	DBLα2	CIDRα1.1	DBLβ12	DBLγ12	DBLδ1	CIDRβ1	Intron			
				1983-9	10%	DBLα0.15	CIDRα2.5	DBLδ1	CIDRβ1	Intron					
				1983-1	9%	DBLα2	CIDRα1.1	DBLβ12	DBLγ4	DBLδ1	CIDRβ1	...			
				1983-7	6%	DBLα1.4	CIDRα1.7	DBLβ1	DBLβ7	DBLγ5	DBLγ11	DBLδ1	CIDRβ4	Intron	
				1983-13	6%	DBLα1.2	CIDRα1.5b	DBLβ6	DBLγ17	DBLγ14	DBLζ5	DBLε4	Intron		
1994	CM	5.8	2	1994-1	23%	Group B/C									
				1994-2	14%	DBLα1.7	CIDRα1.4	DBLβ3	DBLγ13	DBLγ2	DBLγ4	DBLδ1	CIDRβ1	Intron	
				1994-3	10%	DBLα0.9	CIDRα2.2	DBLδ1	CIDRβ1	Intron					
				1994-4	9%	Group A									
				1994-5	7%	Group B/C									
				1994-6	5%	Group B/C									
1995	CM SA RD	3.7	1	1995-2	18%	DBLα0.5	CIDRα2.3	DBLβ10	DBLγ5	DBLδ1	...				
				1995-1	17%	DBLα0.5	CIDRα2.3	DBLδ1	CIDRβ1	...					
				1995-3	9%	DBLα0.19	CIDRα2.9	DBLδ1	CIDRβ3	Intron					
				1995-4	5%	Group B/C									
				1995-5	5%	Group B/C									
				1995-6	4%	Group B/C									
1996	CM SA	4.1	2	1996-1	39%	DBLα0.18	CIDRα5	DBLβ5	DBLγ13	DBLδ3	CIDRγ2	DBLε4	Intron		
				1996-2	13%	DBLα0.15	CIDRα3.1	DBLδ1	CIDRβ1	Intron					
				1996-3	7%	DBLα1.1	CIDRα1.7	DBLβ3	DBLγ2	DBLγ6	DBLδ1	CIDRβ1	Intron		
				1996-4	4%	Group A									
				1996-5	4%	Group B/C									
				1996-6	3%	Group B/C									
2014	CM	8.2	2	2014-1	25%	DBLα1.7	CIDRα1.4	DBLβ3	DBLγ11	DBLγ2	DBLγ4	DBLγ12	DBLδ1	...	
				2014-2	6%	DBLα0.11	CIDRα2.1	DBLβ2	DBLδ1	...					
				2014-3	6%	DBLα0.13	CIDRα2.5	DBLδ1	CIDRβ6	Intron					
				2014-4	6%	DBLα1.4	CIDRα1.3	DBLβ1	DBLγ15	DBLε1	DBLγ8	DBLζ1	DBLε5	Intron	**VAR1**
				2014-5	5%	DBLα2 prediction									
				2014-6	4%	DBLα1.1									
2053	CM Hyper	10.5	2	2053-2	18%	DBLα0.3	CIDRα3.4	DBLδ1	CIDRβ1	...					
				2053-3	15%	DBLα2	CIDRα1.8b	DBLβ12	DBLγ4	DBLδ1	CIDRβ6	Intron			
				2053-1	13%	DBLα1.7	CIDRα1.4	DBLβ3	DBLβ3	DBLγ11	DBLδ1	CIDRβ1	Intron		
				2053-4	9%	DBLα0.13	CIDRα2.3	DBLβ5	DBLδ1	CIDRβ6	Intron				
				2053-5	8%	DBLα2 prediction									
				2053-6	7%	Group B/C									
2065	Hyper	5.1	5	2065-1	22%	DBLα0.8	CIDRα2.6	DBLβ8	DBLδ1	CIDRβ1	Intron				
				2065-3	19%	DBLα1.2	CIDRα1.1	DBLβ12	DBLγ11	DBLγ2	...				
				2065-2	19%	DBLα0.22	CIDRα3.1	DBLβ5	DBLγ5	DBLδ1	CIDRβ1	Intron			
				2065-4	17%	DBLα1.1	CIDRα1.2	...							**VAR1**
				2065-5	7%	Group B/C									
				2065-6	6%	Group B/C									
1712	UM	5.1	5	1712-1	8%	DBLα1.2	CIDRα1.5a	DBLβ6	DBLγ12	DBLδ6	CIDRβ3	DBLβ9	DBLγ11	...	
				1712-2	7%	DBLα2 prediction									
				1712-3	6%	DBLα0.19	CIDRα2.10	DBLβ10	DBLδ1	CIDRβ1	Intron				
				1712-4	6%	DBLα2	CIDRα1.8b	DBLβ12	DBLγ4	DBLδ1	CIDRβ1	Intron			
				1712-5	5%	DBLα0.16	CIDRα3.4	DBLβ8	DBLδ1	CIDRγ12	Intron				
				1712-6	5%	Group B/C									
1702	UM	8	5	1702-1	50%	DBLα2	CIDRα1.1	DBLβ3	DBLγ11	DBLζ4	...				
				1702-2	14%	DBLα0.1	CIDRα3.1	DBLδ1	CIDRβ1	Intron					
				1702-3	12%	DBLα2	CIDRα1.8b	DBLβ12	DBLδ1	CIDRβ1	Intron				
				1702-4	4%	Group A									
				1702-6	4%	DBLα2 prediction									
				1702-5	4%	VAR1									
1678	UM<br>RD Coma †<br>(sepsis)	6.5	2	1678-1	70%	DBLα0.9	CIDRα2.4	DBLδ1	CIDRβ1	DBLγ14	DBLζ5	DBLε4	Intron		
				1678-2	18%	DBLα2	...								
				1678-3	6%	DBLα0.9	CIDRα2.7	DBLγ11	DBLζ5	DBLε4	Intron				
				1678-4	3%	DBLα0.8	CIDRα2.2	DBLδ1	CIDRβ1	Intron					
				1678-5	2%	Group B/C									
				1678-6	1%	Group B/C									
1715	UM	8	5	1715-1	17%	DBLα0.9	CIDRα2.2	DBLδ1	CIDRγ7	Intron					
				1715-2	8%	DBLα1.2	CIDRα1.6b	DBLβ3	DBLγ11	DBLγ4	Intron				
				1715-3	6%	DBLα0.18	CIDRα3.1	DBLβ5	DBLδ1	CIDRβ1	DBLγ3	DBLζ4	Intron		
				1715-4	6%	DBLα0.1	CIDRα3.4	DBLδ1	CIDRβ5	Intron					
				1715-5	5%	Group B/C									
				1715-6	4%	Group B/C									
1878	UM	5.1	5	1878-1	22%	DBLα1.5	CIDRδ1	DBLβ3	DBLγ2	DBLγ9	...				
				1878-2	14%	DBLα0.9	CIDRα2.2	DBLδ1	CIDRγ5	Intron					
				1878-3	11%	DBLα1.7	CIDRα1.7	DBLβ3	DBLβ9	DBLε13	DBLδ1	...			
				1878-4	7%	Group A									
				1878-5	6%	Group A									
				1878-6	5%	Group A									
1886	UM	7.8	3	1886-1	17%	DBLα0.6	CIDRα3.1	DBLβ5	DBLγ18	DBLδ1	CIDRβ5	DBLγ9	Intron		
				1886-2	11%	DBLα0.18	CIDRα5	DBLβ4	DBLγ16	DBLζ4	Intron				
				1886-3	10%	DBLα0.11	CIDRα2.4	DBLδ1	CIDRβ1	Intron					
				1886-4	9%	DBLα0.12	CIDRα4	DBLδ1	CIDRβ1	Intron					
				1886-5	7%	DBLα1.2	CIDRα1.6b	DBLβ7	DBLγ2	DBLγ4	Intron				
				1886-6	5%	Group B/C									
1848	UM	9.9	5	1848-1	27%	DBLα0.6	CIDRα3.1	DBLβ5	DBLγ10	DBLδ1	CIDRβ4	Intron			
				1848-2	22%	DBLα0.15	CIDRα3.2	DBLδ1	CIDRβ1	Intron					
				1848-3	10%	DBLα0.14	CIDRα4	DBLδ1	CIDRβ7	Intron					
				1848-4	7%	DBLα0.23	CIDRα5	DBLβ5	DBLγ16	DBLδ1	CIDRβ1	...			
				1848-5	6%	DBLα0.4	CIDRα6	DBLβ5	DBLγ9	DBLζ2	DBLε4	Intron			
				1848-6	5%	VAR1									
1799	UM<br>RD † (sepsis)	5.1	3	1799-1	55%	DBLα1.6	CIDRγ3	DBLγ7	DBLδ1	CIDRγ6	DBLε13	DBLζ5	DBLε4	Intron	
				1799-2	28%	DBLα1.6	CIDRδ1	DBLγ15	DBLε1	DBLζ3	DBLε6	Intron			
				1799-3	4%	DBLα1.8	CIDRβ2	DBLγ7	DBLε11	DBLζ2	DBLε6	Intron			
				1799-4	4%	Group A									
				1799-5	3%	Group A									
				1799-6	3%	Group B/C									

Figure S2

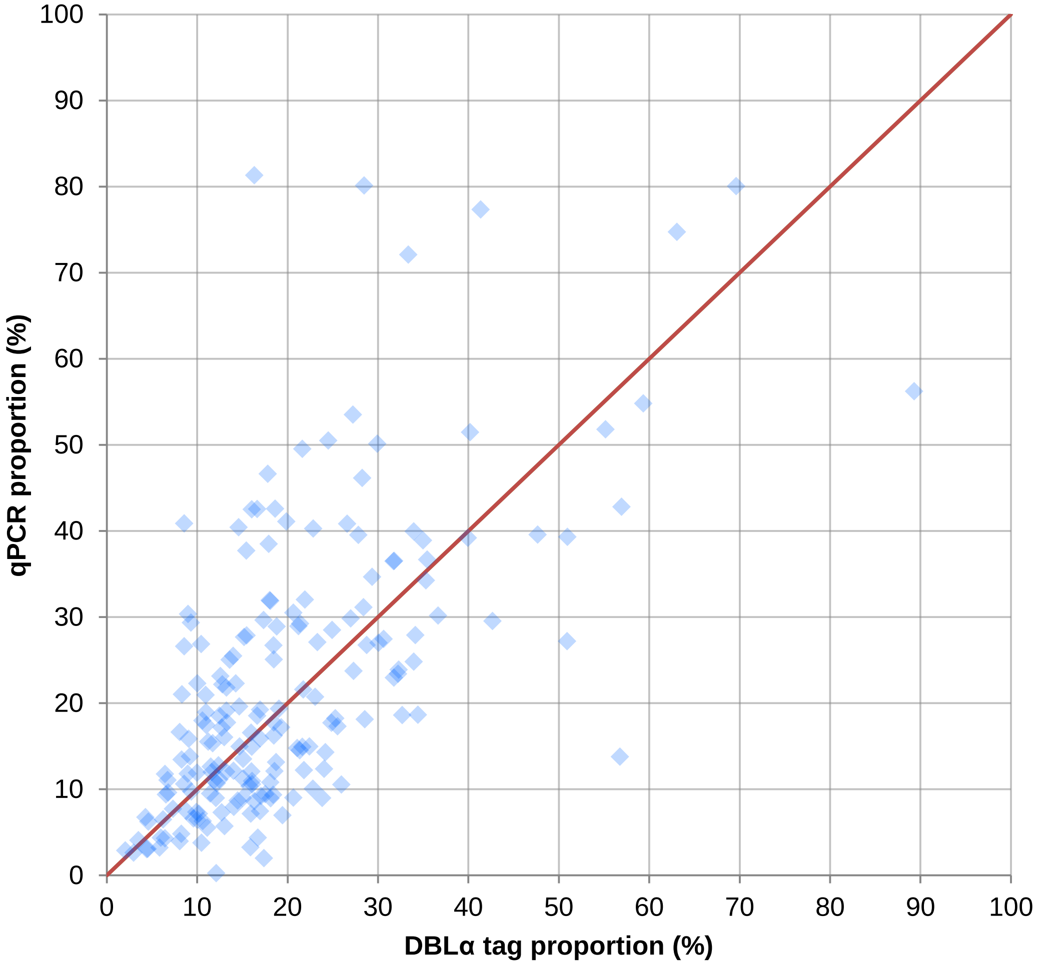

Figure S2: Plot of *var* transcript proportions determined by DBLα-tag anlaysis against qPCR reported transcript proportions. Red line added as a guide for perfect correlation.

Figure S3

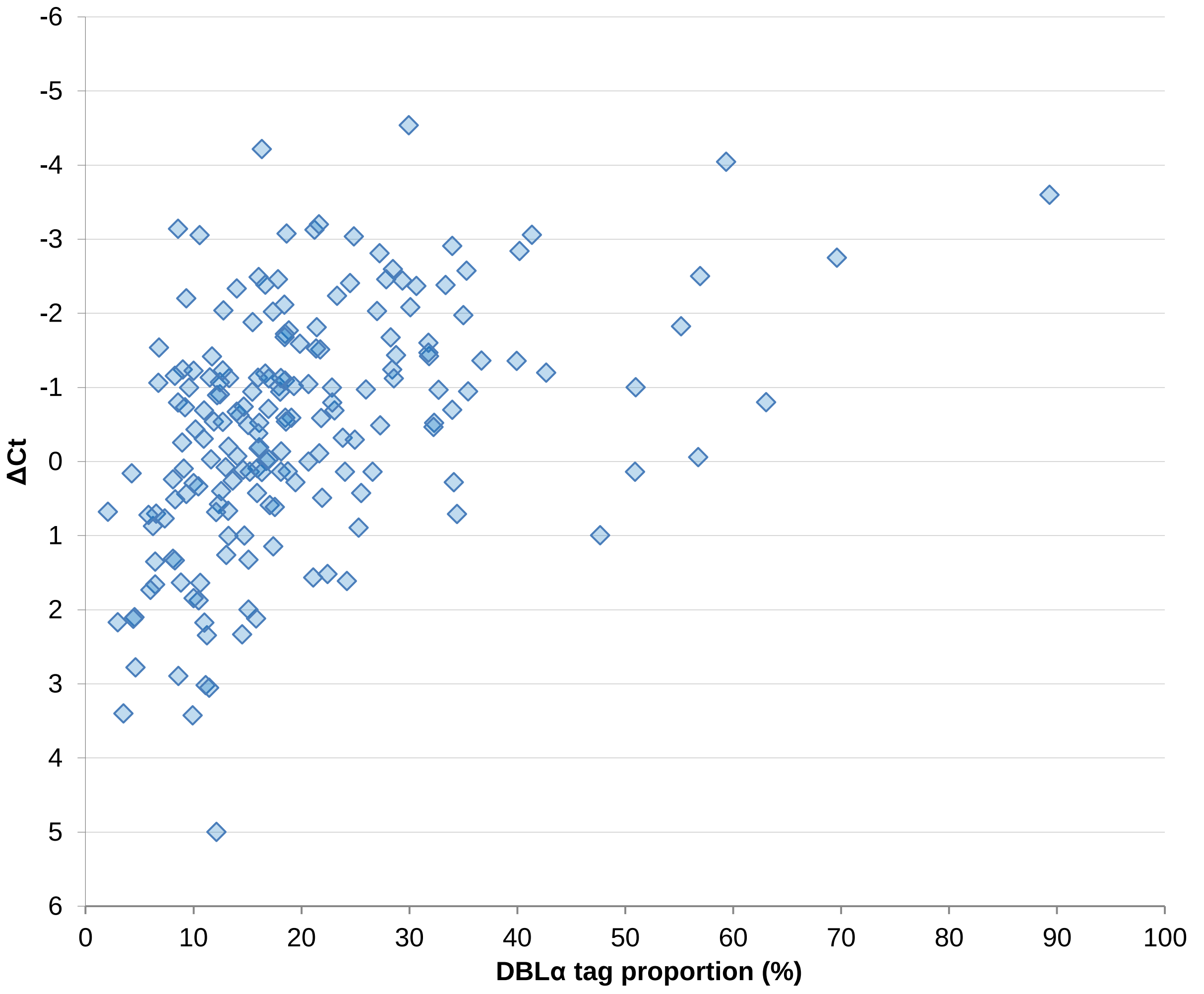

Figure S3: qPCR measured *var* transcript levels (ΔCt relative to endogenous control gene seryl-tRNA synthetase) plotted against the DBLα-tag determined transcript proportion.
