# Supplementary material for: Plasmodium falciparum var genes expressed in children with severe malaria encode CIDRα1 domains
Source: EMBO Mol Med. 2016 Jun 27;8(8):839–50. doi: 10.15252/emmm.201606188 (PMC4967939; doi:10.15252/emmm.201606188)
Supplement: Supplementary file 2 — Expanded View Figures PDF [file EMMM-8-839-s002.pdf]

Expanded View Figures

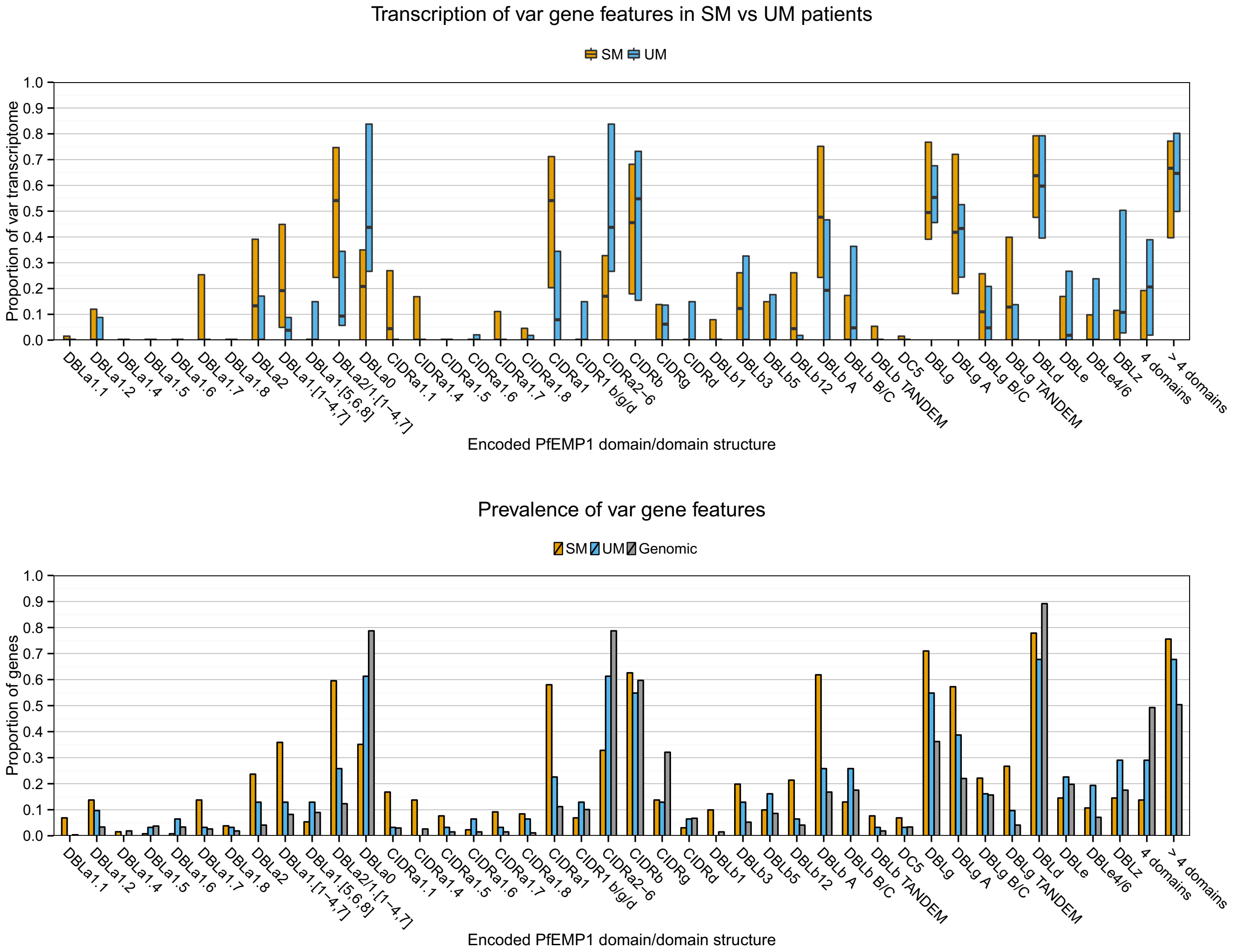

Figure EV1. Prevalence and transcript levels of var genes encoding specified PfEMP1 features.
Upper panel: Patient-wise *var* transcript proportions of encoded domain types/domain compositions, compared by patient group [uncomplicated malaria (UM), n = 8 and severe malaria (SM), n = 36]. Boxplots show median and IQR. Lower panel: Proportion of transcripts/genes encoding specific domain types/domain compositions within patient groups [UM (n = 31 genes), SM (n = 131 genes)] and the average *Plasmodium falciparum* genome [distribution based on seven genomes (n = 268 genes)].

© 2016 The Authors
